# PRKCA Polymorphism Changes the Neural Basis of Episodic Remembering in Healthy Individuals

**DOI:** 10.1371/journal.pone.0098018

**Published:** 2014-05-19

**Authors:** Catherine A. MacLeod, David I. Donaldson

**Affiliations:** 1 Institute of Medical and Social Care Research, Bangor University, Bangor, Gwynedd, United Kingdom; 2 Psychology, School of Natural Sciences, University of Stirling, Stirling, United Kingdom; University of California, San Francisco, United States of America

## Abstract

Everyday functioning relies on episodic memory, the conscious retrieval of past experiences, but this crucial cognitive ability declines severely with aging and disease. Vulnerability to memory decline varies across individuals however, producing differences in the time course and severity of memory problems that complicate attempts at diagnosis and treatment. Here we identify a key source of variability, by examining gene dependent changes in the neural basis of episodic remembering in healthy adults, targeting seven polymorphisms previously linked to memory. Scalp recorded Event-Related Potentials (ERPs) were measured while participants remembered words, using an item recognition task that requires discrimination between studied and unstudied stimuli. Significant differences were found as a consequence of a Single Nucleotide Polymorphism (SNP) in just one of the tested genes, PRKCA (rs8074995). Participants with the common G/G variant exhibited left parietal old/new effects, which are typically seen in word recognition studies, reflecting recollection-based remembering. During the same stage of memory retrieval participants carrying a rarer A variant exhibited an atypical pattern of brain activity, a topographically dissociable frontally-distributed old/new effect, even though behavioural performance did not differ between groups. Results replicated in a second independent sample of participants. These findings demonstrate that the PRKCA genotype is important in determining how episodic memories are retrieved, opening a new route towards understanding individual differences in memory.

## Introduction

Episodic remembering is fundamental to a healthy high-quality life, supporting both day-to-day functioning and creating a coherent sense of self [1]. Episodic memory is also extremely fragile, declining with age [2], and easily disrupted by diseases such as dementia [3], depression [4] and schizophrenia [5]. Attempts to diagnose and treat memory problems are however complicated by individual variation in the timing and severity of symptoms [6]. One possible cause of such variability is an individual’s genotype. In both clinical [7] and healthy populations [8] genetic variability has been found to be a significant source of variation in cognitive abilities [9], including episodic memory, where evidence from twin studies suggests that heritability accounts for between 30% and 60% of variability in performance [8]. In addition to evidence of differences in behaviour [10], a number of functional Magnetic Resonance Imaging (fMRI) studies have shown that genetic variation can influence brain activity associated with memory [11]–[16]. Despite the grounding of many investigations of memory decline in the current understanding of episodic memory in healthy young adults, the full consequences of these genetic differences remain unknown, particularly in those whose memory is intact.

Whilst fMRI studies provide valuable information regarding which neural structures are activated during memory retrieval, the use of the hemodynamic response to measure neural activity results in a 1 to 2 second lag between the neural event and the information recorded whilst the vascular system responds to these changes in the brain. In contrast, measuring the electrophysiological activity of neurons from electrodes placed on the scalp, and generating Event-Related Potential (ERPs) to events of interest, provides real-time information about the neural activity associated with that event. Despite the superior temporal quality of ERPs there does not appear to be any research looking at the relationship between ERP correlates of episodic memory retrieval and genetic polymorphisms. In light of the quick onset and duration of retrieval processes the question arises as to whether there is genetic variability in neural activation associated with memory retrieval, and if so, whether such variability is time-specific?

Here we use ERPs to investigate genetic variability in the neural basis of remembering, targeting polymorphisms (in genes *ADCY8* [*17*], *APOE* [*16*], *BDNF* [*11*], *COMT* [*18*], *KIBRA* [*13*], *PRKACG* [*17*] and *PRKCA* [*17*]) previously linked to episodic memory across patient and genome-wide association studies. One hundred and twenty nine healthy young adults provided saliva samples (using Oragene OG-100 vials, DNA Genotek Inc), allowing DNA to be extracted and genotyped at the Wellcome Trust Clinical Research Facility, Edinburgh. To avoid the possibility that mental health issues could act as a confound [19] participants completed a Psychiatric Diagnostic Screening Questionnaire [20], leading to the exclusion of 43 participants, with data from 86 participants analysed.

## Results

Brain activity was measured using scalp recorded ERPs, providing high temporal resolution neural data that allows genetic effects to be linked to distinct stages of memory processing. ERPs were recorded while participants performed a visual item recognition memory test, using common six-letter nouns and verbs as stimuli. Participants studied 50 words, subsequently discriminating them from 50 additional randomly inter-mixed unstudied words at test (responding ‘old’ or ‘new’). Memory performance was excellent overall, with 72% of old and 82% of new words receiving correct responses, reflecting high levels of discrimination [21] (Pr mean = 0.53, s.d. = 0.17; Br mean = 0.39, s.d. = 0.16). An additional two participants were excluded from further analysis on the basis of low discrimination (Pr<0.2), ensuring that neural activity was examined only for participants who were unquestionably remembering, leaving 84 participants for inclusion in the genetic analysis.

For each participant average ERP waveforms were generated for correctly recognized old (Hits) and correctly rejected new (CRs) words. Averaged across participants the resulting ERP ‘old/new effect’ (i.e., the difference between Hits and CRs) is a reliable well-established neural correlate of retrieval success [22], consistent with multi-process models of episodic memory [23]. Critically, ERP old/new effects capture two core temporally distinct stages of retrieval: an early (300–500 ms post-stimulus) positivity over frontal scalp has been linked with familiarity [24] and conceptual priming [25], whereas a later onsetting (500–700 ms post-stimulus) positivity over left parietal scalp reflects recollection based retrieval [26]. Focusing on these two existing ERP old/new effects constrains our analysis strategy, providing strong *a priori* hypotheses about the pattern of neural activity expected during episodic retrieval.

**Figure 1:**
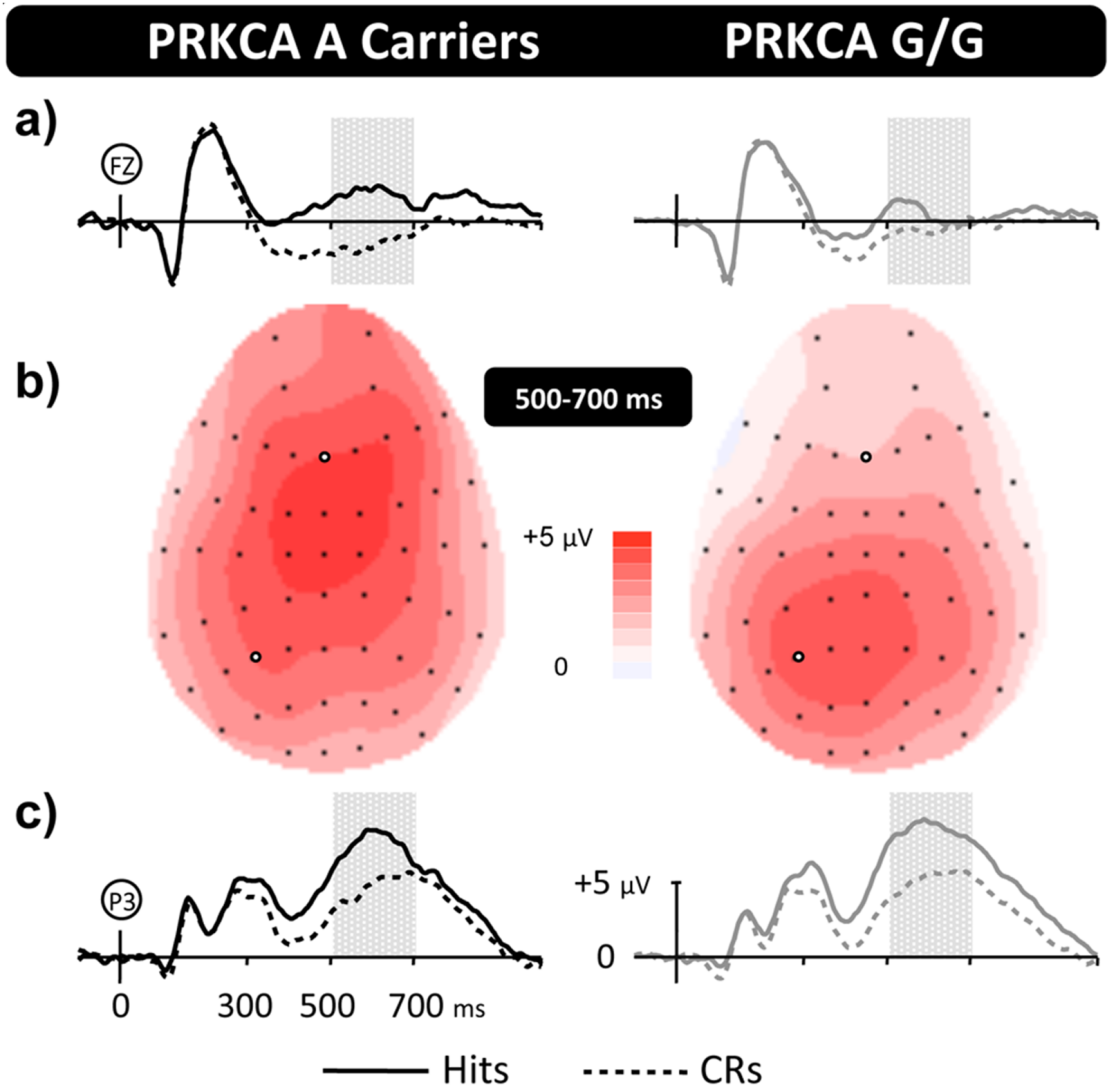
Distinct patterns of memory related brain activity for PRKCA polymorphisms. Grand-average ERP old/new effects for PRKCA genotypes at representative Frontal (**a**) and Left-Parietal (**c**) electrodes, along with topographic maps (**b**) illustrating the distribution of old/new effects from 500–700 ms. The vertical scale indicates electrode amplitude (microvolts) and the horizontal scale change in time (milliseconds), with markers indicating the 500–700 ms window where significant results were found. The colour scale indicates Hit-CR difference size (microvolts). For both groups Hit ERPs are more positive going than CR from ∼300 ms post-stimulus onset (0 ms), reconverging by epoch end. Topographically dissociable maxima are evident across groups: parietally focused for G/G carriers and frontally focused for A carriers.

ERP data was examined separately for each polymorphism, collapsing polymorphisms of n<16 where possible, and excluding polymorphisms where not (as outlined in Table 1). The number of participants in the final analysis are as follows: n = 72 for polymorphism rs3249 in gene ADCY8, n = 78 for the combined polymorphisms of rs7412 and rs429358 in gene APOE, n = 84 for polymorphism rs6265 in gene BDNF, n = 84 for polymorphism rs4650 in gene COMT, n = 80 for polymorphism rs17070145 in gene KIBRA, n = 84 for polymorphism rs3730386 in gene PRKACG, and n = 82 for polymorphism rs80764995 in gene PRKCA. Analysis compared ERP old/new difference waveforms during early (300–500 ms) and late (500–700 ms) stages of retrieval. An initial global omnibus ANOVA was performed for each polymorphism, with a between-subjects factor of genotype and a within-subjects factor of electrode (35 sites covering the scalp including locations F/FC/C/CP/P at electrode sites 1/3/5/z/2/4/6; see Figure S1). Results are presented in Table 2, showing a single significant genotype by electrode interaction for PRKCA, during the late time window, with no significant genotype by electrode interactions found for the earlier 300–500 ms time window. Figure 1 shows group average ERPs for A and G/G variants of PRKCA, along with topographic scalp maps illustrating the distribution of old/new effects from 500–700 ms (Figure S2 shows equivalent maps for all other polymorphisms).

**Table 1:** Candidate genes included in the global omnibus ANOVA.

Gene	Hardy-Weinberg equilibrium (n = 129)	Polymorphism	N (frequency)^1^
**ADCY8**	*X* ^2^ = 1.72, p = 0.19	*A/A*	*11 (13%)*
*(rs263249)*		A/G	40 (48%)
		G/G	32 (38%)
**APOE**	*X* ^2^ = 3.06, p = 0.08 *(rs7412)*	ε2 carriers	19 (23%)
*(rs7412 &*	*X* ^2^ = 1.32, p = 0.25 (*rs429358)*	ε3/ε3	41 (49%)
*rs429358)*		ε4 carriers	18 (21%)
**BDNF**	*X* ^2^ = 2.98, p = 0.08	**A/A**	**1 (1%)**
*(rs6265)*		**A/G**	**28 (33%)**
		G/G	55 (66%)
**COMT**	*X* ^2^ = 0, p = 0.98	A/A	23 (27%)
*(rs4680)*		A/G	42 (50%)
		G/G	19 (23%)
**KIBRA**	*X* ^2^ = 0.97, p = 0.32	C/C	35 (42%)
*(rs17070145)*		**C/T**	**38 (45%)**
		**T/T**	**7 (8%)**
**PRKACG**	*X* ^2^ = 0.49, p = 0.48	C/C	58 (69%)
*(rs3730386)*		**C/G**	**24 (29%)**
		**G/G**	**2 (2%)**
**PRKCA**	*X* ^2^ = 0.51, p = 0.47	**A/A**	**4 (5%)**
*(rs8074995)*		**A/G**	**20 (24%)**
		G/G	58 (69%)

1 Only variants with n>16 were analysed, with variants not included in the analysis presented in italic, and variants collapsed into a single carrier group presented in bold.

**Table 2:** Statistical results of the global omnibus ANOVA.

Genotype	300–500 ms	500–700 ms
*ADCY8* (A/G v. G/G)	F(3,184) = 0.775, p = 0.494	F(3,201) = 1.219, p = 0.304
*APOE* (ε2 v. ε3)	F(3,158) = 1.353, p = 0.261	F(3,160) = 1.560, p = 0.204
*APOE* (ε2 v. ε4)	F(3,91) = 1.88, p = 0.147	F(3,98) = 1.434, p = 0.239
*APOE* (ε3 v. ε4)	F(3,157) = 0.221, p = 0.867	F(3,175) = 0.165, p = 0.923
*BDNF* (A Carriers v. G/G)	F(3,226) = 0.181, p = 0.896	F(3,241) = 0.206, p = 0.889
*COMT* (A/A v. G/G)	F(3,118) = 1.125, p = 0.341	F(3,111) = 0.319, p = 0.796
*COMT* (A/G v. A/A)	F(2,157) = 0.157, p = 0.895	F(3,189) = 0.342, p = 0.795
*COMT* (A/G v. G/G)	F(3,162) = 1.112, p = 0.343	F(3,175) = 1.277, p = 0.284
*KIBRA* (C/C v. T Carriers)	F(3,215) = 0.844, p = 0.463	F(3,229) = 0.434, p = 0.725
*PRKACG* (C/C v. G Carriers)	F(3,229) = 1.339, p = 0.263	F(3,242) = 0.362, p = 0.777
*PRKCA* (A Carriers v. G/G)	F(3,224) = 1.261, p = 0.289	**F(3,247) = 3.945, p = 0.008** *

*Significant differences (p<0.05) are highlighted in bold and starred.

**Figure 2:**
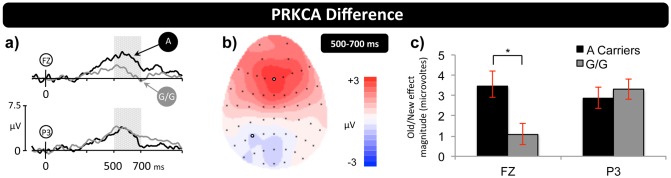
PRKCA polymorphisms elicit differences over frontal scalp electrodes. (**a**) Grand-average ERP difference waveforms (Hits-CRs) for PRKCA A and G/G carriers at electrodes Fz and P3, showing equivalent activity over parietal scalp, but greater activity for A carriers over frontal scalp. (**b**) Topographic map from 500–700 ms illustrating the distribution of the difference between PRKCA A and G/G old/new effects. (**c**) Histogram of mean old/new effect magnitude (in microvolts) at midline-frontal (Fz) and left-parietal (P3) electrodes, from 500–700 ms, for each PRKCA genotype. Statistical analysis confirms significant gene-dependent differences in activity at frontal but not parietal sites. Scale bars as in Figure 1.

To further characterise the phenotypic difference in the pattern of brain activity found for each PRKCA group we carried out regional analysis of the 500–700 ms data. ANOVA directly compared the old/new effects (i.e., subtraction waveforms) using a between-subjects factor of genotype, and within-subjects factors of location (F/FC/C/CP/P), hemisphere (L/R), and electrode (I/M/S) including locations F/FC/C/CP/P at electrode sites 1/3/5 on the left and 2/4/6 on the right. A significant genotype by location interaction (F(1,94) = 6.9, p = 0.007) reflects the more anteriorly distributed old/new effect seen for A carriers compared to homozygous G carriers. Importantly, topographic analysis using rescaled data (normalised to remove amplitude differences) also revealed a significant genotype by location interaction (F(1,94) = 6.9, p = 0.007), confirming that the pattern of activity differs in distribution not just magnitude. Figure 2 shows ERP difference waveforms for A and G/G groups, along with a scalp map illustrating the difference between groups.

Topographically distinct memory related ERP effects have been reported previously as a function of sex, e.g., in memory for faces [27], introducing a potential confound here. We therefore re-examined the group data as a function of sex (with categorisation based on a self report of either male or female), finding that the heterozygous PRKCA group included a significantly larger proportion of males than the homozygous group (50% vs. 26% respectively, Fisher's exact test yields p = 0.042). Given this significant difference we reanalyzed the ERP data for the 500–700 ms time window, introducing sex as an additional between-subjects factor. Analysis revealed no significant main effects of sex or interactions involving sex, for either the omnibus (all p>0.2) or regional (all p>0.1) analyses, confirming that sex differences did not influence the results.

Regional analysis suggests that A allele carriers exhibit additional positivity over frontal electrodes (Figure 2). To confirm the focus of effects, t-tests were carried out on data from virtual frontal (F1, Fz, F2) and left parietal (P5, P3, P1) electrodes, revealing a significant difference over frontal (mean difference = 2.23 µV; t(80) = 2.349, p = 0.021) but not parietal (mean difference = −0.36 µV; t(80) = −0.438, p = 0.662) sites. Analysis of genetic variants inherently relies on a comparison of two unequally sized groups (A carriers = 24, G/G = 58), potentially introducing biased sampling and violations of homoscedasticity. Consequently a bootstrap analysis [28] was carried out on data from the virtual frontal electrode. Re-averaging the G/G data 500 times, using random subsets of 24 participants each time, confirmed the significant gene-cognition effect (mean difference of 2.23 µV, bootstrap 95% confidence intervals of 2.17 µV and 2.29 µV, p<0.001).

The difference between A and G/G carriers could, in principle, reflect differential engagement of the retrieval mechanisms that support episodic remembering (i.e., recollection, familiarity and priming) [23]. Equally, the difference could reflect greater reliance on monitoring and control processes, such as those located within the frontal lobes [26]. To assess these possibilities we examined behavioral measures as a function of gene, on the basis that changes of this type would be expected to alter either the timing or accuracy of retrieval [29]. Analysis of discrimination accuracy (A mean = 0.54, s.d. = 0.18; G/G mean = 0.53, s.d. = 0.16), decision bias (A mean = 0.39, s.d. = 0.17; G/G mean = 0.38, s.d. = 0.16), and response times (HIT: A mean = 852 ms, s.d. = 187 ms; G/G mean = 811 ms, s.d. = 109 ms, and CR: A mean = 908 ms, s.d. = 188 ms; G/G mean = 891 ms, s.d. = 136 ms) all revealed no significant difference between groups (independent sample t-tests, all p>0.2), suggesting that carriers of the rare PRKCA variant actually exhibit an atypical pattern of neural activity when remembering.

The use of multiple comparisons across seven different genes raises the serious possibility that the PRKCA result may be a statistical false positive [30]. We therefore calculated a strict Bonferroni adjusted alpha level of 0.0023 for each test (0.05/22: 11 polymorphism comparisons over two time-windows); with this adjustment the PRKCA global omnibus ANOVA (F(3,247) = 3.945, p = 0.008) does not reach significance. Although the Bonferroni correction is arguably overly conservative, the failure to survive a correction for multiple comparisons led us to conduct a targeted independent replication, focusing specifically on the PRKCA rs8074995 polymorphism and ERP activity in the 500–700 ms time-window. Sixty three participants (PRKCA frequencies A/A = 2, A/G = 14, G/G = 44, undetermined = 3; Hardy-Weinberg equilibrium χ^2^ = 0.43, p = 0.51) completed two study/test blocks using two hundred 5–7 letter words (50 old words and 50 new words per block). Analysis of all 63 participants indicates that overall task performance mirrored that of Experiment One, with 70% of old and 82% of new words receiving correct responses, reflecting high discrimination (Pr mean = 0.51, s.d. = 0.21; Br mean = 0.39, s.d. = 0.23). The task and procedures were identical to that used previously, except that after each ‘old’ response participants made a Remember (R), Know (K) or Guess (G) judgement [31]. The R/K/G distinction allows recollection (R = 59%) based responses to be identified and examined in isolation, excluding ‘no-recollection’ trials (a combination of familiarity (K = 31%) and guessing (G = 10%)). As previously, participants were excluded from further analysis if they had undetermined genotype, exhibited poor memory, or had insufficient artifact-free trials in any response category (3, 4 and 29 participants respectively) leaving 27 participants. An additional four participants were excluded because they had contributed to the original sample, leaving 23 participants contributing ERP data (10 A carriers and 13 homozygous G carriers). Performance for the sub-sample of 23 participants reflected that of the full sample, with 71% of old and 81% of new words receiving correct responses, again reflecting high discrimination (Pr mean = 0.52, s.d. = 0.13; Br mean = 0.39, s.d. = 0.20) and high levels of recollection (R = 56%, K = 31%, G = 13%). ERP Analysis focused on neural activity associated with recollection (Remember minus CR difference waveforms), between 500–700 ms post-stimulus, across genotypes.

**Figure 3:**
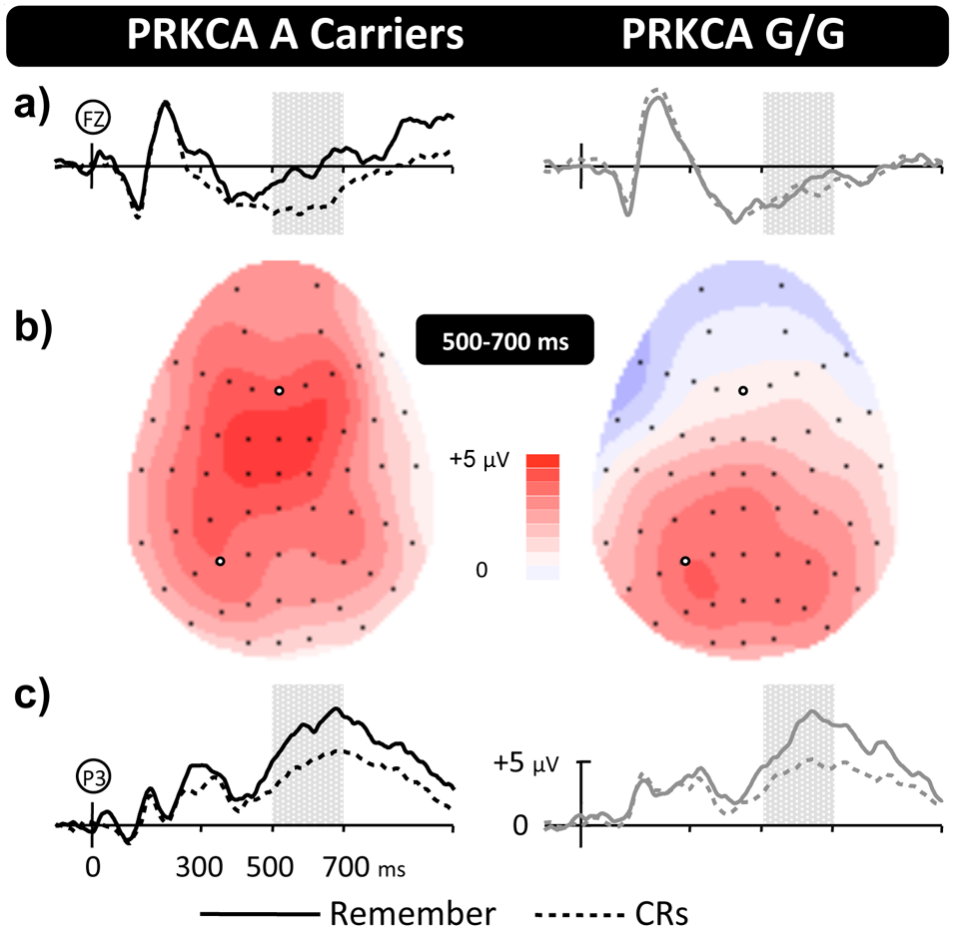
Replication of PRKCA dependent patterns of memory related brain activity. Grand-average ERP old/new effects for each PRKCA genotype from Experiment Two are shown at Frontal (**a**) and Left-Parietal (**c**) electrode sites, along with topographic maps (**b**) illustrating the distribution of effects (Remember-CR) between 500–700 ms. The pattern of activity replicates initial findings (see Figure 1) with topographically dissociable maxima across polymorphisms, showing a parietal focus for the common G/G group but a frontal focus for the rarer A group. Data shown as in Figure 1.

Group average ERPs for A carriers and G/G carriers are presented in Figure 3, alongside maps showing the distribution of activity. A global ANOVA (35 sites, including locations F/FC/C/CP/P at electrode sites 1/3/5/z/2/4/6) revealed a significant genotype by electrode interaction (F(3,67) = 3.99, p = 0.010). Regional analysis revealed a significant genotype by location interaction (F(1,27) = 7.00, p = 0.009), indicating greater activity over anterior electrodes for A carriers compared to G/G carriers. An additional genotype by hemisphere by electrode interaction (F(2,36) = 5.59, p = 0.011) indicates that the difference between genotypes is larger over the left than right hemisphere, particularly at inferior electrodes. Importantly, topographic analysis revealed significant genotype by location (F(1,27) = 6.81, p = 0.010), and genotype by hemisphere by electrode (F(2,36) = 5.88, p = 0.008) interactions, confirming the presence of a qualitative difference in retrieval-related brain activity. As in the original experiment no significant (all p>0.1) behavioural differences were found between groups across measures of discrimination accuracy (A mean = 0.49, s.d. = 0.12; G/G mean = 0.54, s.d. = 0.14), decision bias (A mean = 0.34, s.d. = 0.17; G/G mean = 0.43, s.d. = 0.21), or response times (HIT: A mean = 1119 ms, s.d. = 151 ms; G/G mean = 1230 ms, s.d. = 238 ms, and CR: A mean = 1123 ms, s.d. = 270 ms; G/G mean = 1337 ms, s.d. = 355 ms), nor in the additional recollection rate data (A mean = 0.54, s.d. = 0.11; G/G mean = 0.58, s.d. = 0.11). In short, we replicated the original findings, with carriers of the PRKCA A allele exhibiting more frontally distributed ERP effects during retrieval than homozygous G carriers.

## Discussion

The results presented indicate differences in memory related neural activity as a function of PRKCA polymorphism rs8074995. The rs8074995 SNP occurs in the PRKCA gene that encodes the alpha isoform of Protein Kinase C (PKCα), which is known to be involved in the trafficking of N-Methyl-D-Aspartate (NMDA) receptors [32], mediating long-term potentiation and synaptic plasticity [33]. The discovery of PRKCA associated differences in retrieval-related brain activity highlights the important role of PKCα in episodic memory. A link between possession of the PRKCA homozygous A variant and reduced episodic recall has been previously identified in people with schizophrenia who exhibit cognitive impairment [19]. Given the importance of neural dys-connectivity for schizophrenia [34], the present findings suggest that the consequences of abnormal integration between brain regions for memory should be greater for A carriers than G/G carriers, due to the reliance on a wider neural network for successful memory retrieval.

The present findings provide clear evidence of a link between variability in PRKCA polymorphism and variability in the neural basis of episodic memory. As with any genetic association study it remains possible that the SNP (rs8074995) has high linkage disequilibrium with a polymorphism at another loci, which is itself driving the neural difference. In addition, although we replicated our results in an independent sample, the relatively small size of our participant groups merit caution (small samples necessarily increase the risk of false positive results [35]), suggesting that a further, larger-scale, replication is essential. Regardless, our findings provide clear evidence that the neural basis of episodic memory retrieval can vary across participants, and suggests that the rs8074995 SNP plays a role in memory retrieval, providing an effective marker of individual differences in the neural basis of episodic memory.

Our results show that genetic differences in neural activity were limited to 500–700 ms after stimulus presentation. During the 500–700 ms post-stimulus time-window participants with the common G/G polymorphism exhibited typical left parietal old/new effects, reflecting recollection of contextual information from memory, a finding that was corroborated by isolating Remember responses in Experiment Two. More importantly, the high temporal resolution of the ERP data reveals that A carriers did not exhibit differences in the timing or magnitude of the left parietal effect *per se*. Rather, additional frontal activity was present during the same stage of retrieval, reflecting the engagement of an extended network of brain regions in A carriers. One potential interpretation of our findings is that A carriers exhibit additional frontally mediated strategic monitoring and decision processes [36]. Typically, however, the frontal ERP effects associated with post-retrieval processing exhibit a strong right-side hemispheric asymmetry, and are maximal in size between 1 and 2 seconds post-stimulus – neither of which is true of the old/new effects reported here. In addition, interpretations that rely on claims of additional post-retrieval processing also predict differences in behaviour (e.g., changes in reaction time) – which are not present in the data reported here. Taken together, therefore, the data suggests that recollection operates differently in the two PRKCA groups, a finding that fits well with recent demonstrations that the neural correlates of recollection can vary within individuals depending on the type of material being remembered [37], [38] reflecting different processing demands across material. Whether individuals with the rare PRKCA variant also differ in how they respond to the processing demands imposed by different materials remains to be seen. If such differences do exist, they would suggest that changing *how* you retrieve actually alters *what* you are predisposed to retrieve.

Critically, the results presented here highlight important individual variations in the neural activity associated with episodic memory retrieval, differences that are typically overlooked and ignored. Healthy ‘outliers’, such as PRKCA A Carriers, may provide insight into the associations made between neural activity and specific cognitive processes as to whether such associations are indeed functional, or if they are simply a co-varying or down-stream consequence of other functional activity. For example, the link between PKCα and plasticity suggests that the retrieval-related differences evident here may themselves reflect the consequence of changes occurring during the encoding and storage of memories, a possibility that warrants further investigation. These data also highlight the need for greater attention to individual variability across theories of episodic memory - demanding more sophisticated and nuanced models than currently exist.

In summary, the presented results reveal differing patterns of retrieval related neural activity dependent on PRKCA (rs8074995) polymorphism, but independent of behavioural performance. Eight polymorphisms were tested, with differences in neural activity only evident for the PRKCA polymorphism and restricted to the later 500–700 ms time-window, typically associated with recollection of contextual information. The results therefore provide evidence for a role of PRKCA in the way we retrieve episodic memories, and specifically in the way we recollect. Furthermore, the existence of gene dependent changes in the underlying neural activity associated with episodic memory retrieval in healthy young participants, highlights individual variation in the way we retrieve memories, questioning the generalisability of current interpretations of the relationship between neural activity and episodic memory retrieval.

## Materials and Methods

### Ethics Statement

Ethical approval for the study was received from the University of Stirling Psychology Ethics Board. All participants gave written informed consent to participate in the study and were fully debriefed upon completion. Participants aged over 16 years were considered to have legal capacity in accordance with the Age of Legal Capacity (Scotland) Act 1991.

### Participants, Stimuli, Procedure

We carried out two independent memory studies. Participants in both Experiment One and Experiment Two were healthy, right-handed, native English speakers, recruited from the University of Stirling, Scotland and were reimbursed for their participation with course credits or at a rate of £5 per hour in Experiment One and £7.50 in Experiment Two. Participants were aged 17–35 years in Experiment One and aged 18–28 years in Experiment Two, and all reported normal/corrected to normal vision; no history of colour blindness, hearing difficulties, dyslexia, neurological problems, brain injury, CNS infection, drug or alcohol abuse, and had not or were not currently receiving treatment for a psychological illness. Participants completed a series of cognitive, neuropsychological and psychometric assessments, the majority of which are not reported here. Although the studies were carried out independently, experimental sign up procedures allowed participants to take part in both experiments; we therefore excluded four participants from Experiment Two on the basis that they had already taken part in Experiment One.

Memory was tested using a PC, with Psychology Software Tools five-button response box and software (Eprime 1.1). For Experiment One, 100 medium frequency six letter words (10–13 occurrences per million [39]) were sorted alphabetically and allocated alternately to two lists, allowing counterbalancing of studied/unstudied status (stimulus order was randomised for each participant). Words were presented for 1000 ms in white 18-point bold Courier New font (black background), proceeded by a 2000 ms cross-hair. Fifty words were presented during study and 100 words during test (fifty old and fifty new) with a one-minute break separating the two phases, during which participants were instructed to relax and rest their eyes. At test participants responded ‘old’ or ‘new’ as quickly and accurately as possible, with responses triggering the next trial. Responses were made with left and right index fingers (counterbalanced across participants). Markedly fast (<300 ms) and slow (>twice the mean) responses were excluded from analysis (mean = 1%). Experiment Two replicated Experiment One with a new set of words that were five to seven letters in length. A secondary Remember/Know/Guess judgment was added at test to ‘old’ judgments, with responses triggering the next trial. ‘Remember’ and ‘Know’ responses were made with left and right index fingers (counterbalanced across participants) and ‘Guess’ responses always made with the center button.

### EEG Acquisition and Analysis

EEG was acquired using Neuroscan amplifier (SynAmps^2^) with electrode caps (Quickcaps) and software (Aquire/Edit 4.3/4.4). Data was recorded from 62 Ag/AgCl electrodes conforming to the International 10–20 System of electrode location. Impedances were kept below 5 KΩ and data was digitised at a rate of 250 Hz, sampling at 4 ms/point, and a band-pass filter of 0.1–40 Hz was used to attenuate both high and low frequencies. Signals were amplified with a gain of 2010.

EEG data was re-referenced off-line to linked-mastoids. Epochs of 1200 ms (−100 to 1100 ms) were extracted from EEG, time-locked to stimulus onset (0 ms), with 100 ms preceding stimulus onset used as a baseline. Eye-blinks were removed using the ocular artifact reduction procedure in Neuroscan Edit software (version 4.3) and trials where drift was greater than ±75 µV or where the signal exceeded ±100 µV on any electrode were excluded. Data was smoothed using a rolling average, over a successive 5 point kernel. Average ERPs were formed for each participant (Experiment One trials: Hit/CR mean = 29/32 respectively; min/max = 16/45; mean trials rejected = 16%; Experiment Two: R/CR mean = 32/61; min/max = 18/79; mean trials rejected = 22%). Mean old/new effect amplitude (Experiment One: Hit-CR; Experiment Two: R-CR) was calculated for each genotype, analysed using ANOVA (Greenhouse-Geisser corrected for non-sphericity as appropriate), with topographic differences assessed following max/min rescaling [40].

### SNP Genotyping

Saliva samples were collected using Oragene OG-100 DNA collection vials (DNA Genotek Inc) in accordance with Oragene guidelines. DNA was extracted by Welcome Trust Clinical Research Facility Edinburgh from saliva using Oragene Purifier OG-L2P-5 and quantified using Picogreen dye. SNP genotyping was conducted using an Applied Biosystems 7900HT Fast Real-Time PCR system, with Taqman SNP assays rs6265, rs17070145, rs7412, rs429358, rs4680, rs263249, rs8074995, rs3730386 (Applied Biosystems).

## Supporting Information

Figure S1 **Schematic illustration of electrode montage.** EEG was recorded from 62 electrodes arranged according to the extended International 10–20 system. Electrodes are displayed as if looking down on the top of the head, with the nose at the top of the oval. The figure illustrates the pattern of electrodes used in statistical analysis - all 35 electrodes employed in the global omnibus ANOVA are enlarged. The allocation of these electrodes into factors for regional analysis is also indicated, using location, hemisphere and electrode markers.
(TIF)

Figure S2 **Retrieval related brain activity is unaffected by polymorphisms of ADCY8, APOE, BDNF, COMT, KIBRA, PRKACG.** Topographic maps depicting the distributions of the old/new effects (Hits minus CRs) in the 500–700 ms time-window for all genes included in the global omnibus ANOVA that failed to reveal significant genotype differences. As evident from the analysis there is minimal difference between genotypes, with carriers of both common and rare variants of each polymorphism exhibiting the typical left parietal distribution reported in the literature. The scale bar indicates the size of the old/new difference in microvolts.
(TIF)
