# Supplementary figures and images for: PRKCA Polymorphism Changes the Neural Basis of Episodic Remembering in Healthy Individuals
Source: PLoS One. 2014 May 19;9(5):e98018. doi: 10.1371/journal.pone.0098018 (PMC4026476; doi:10.1371/journal.pone.0098018)

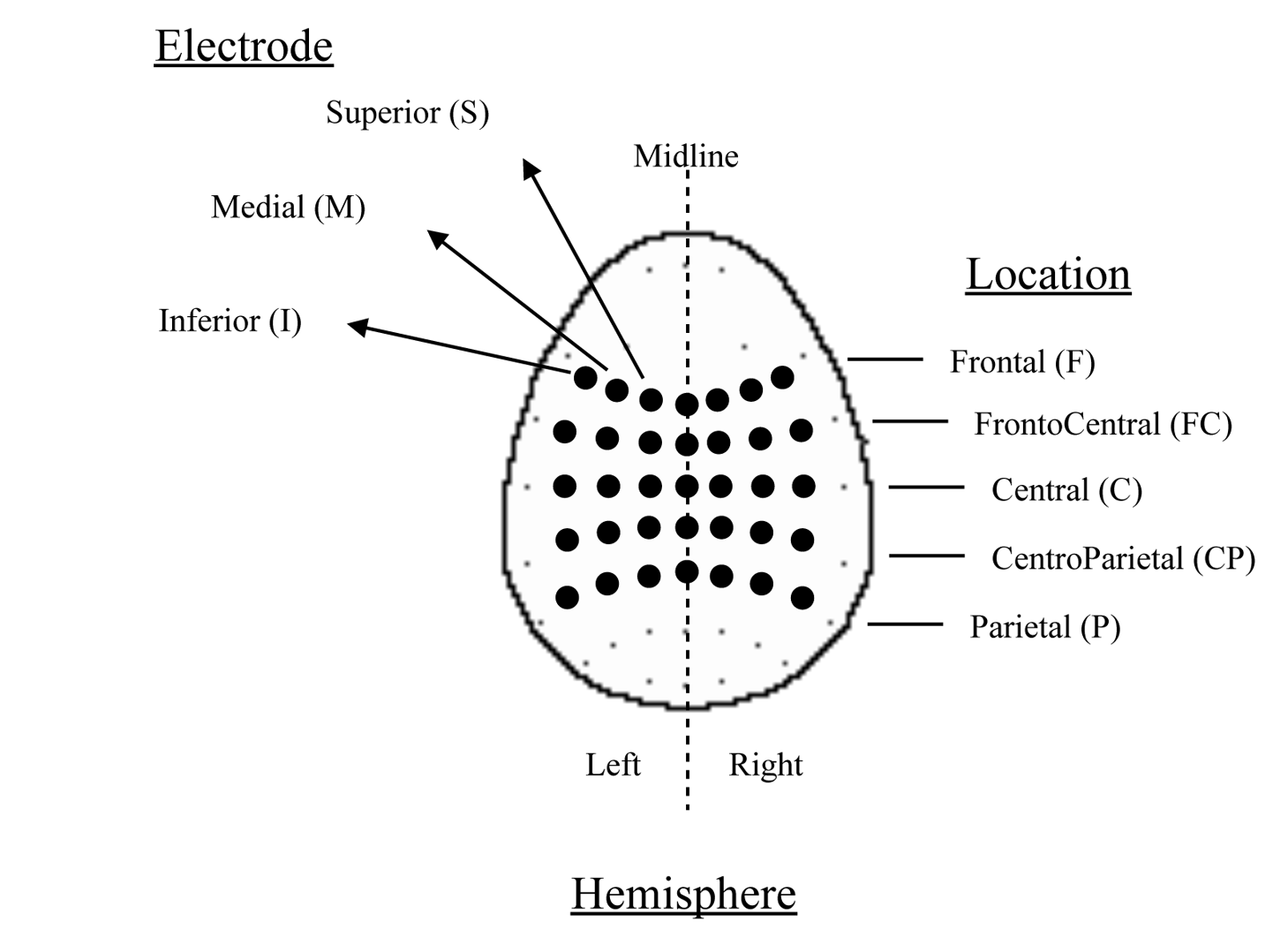

Supplement: Figure S1 — Schematic illustration of electrode montage. EEG was recorded from 62 electrodes arranged according to the extended International 10–20 system. Electrodes are displayed as if looking down on the top of the head, with the nose at the top of the oval. The figure illustrates the pattern of electrodes used in statistical analysis - all 35 electrodes employed in the global omnibus ANOVA are enlarged. The allocation of these electrodes into factors for regional analysis is also indicated, using location, hemisphere and electrode markers. (TIF) [file pone.0098018.s001.tif]

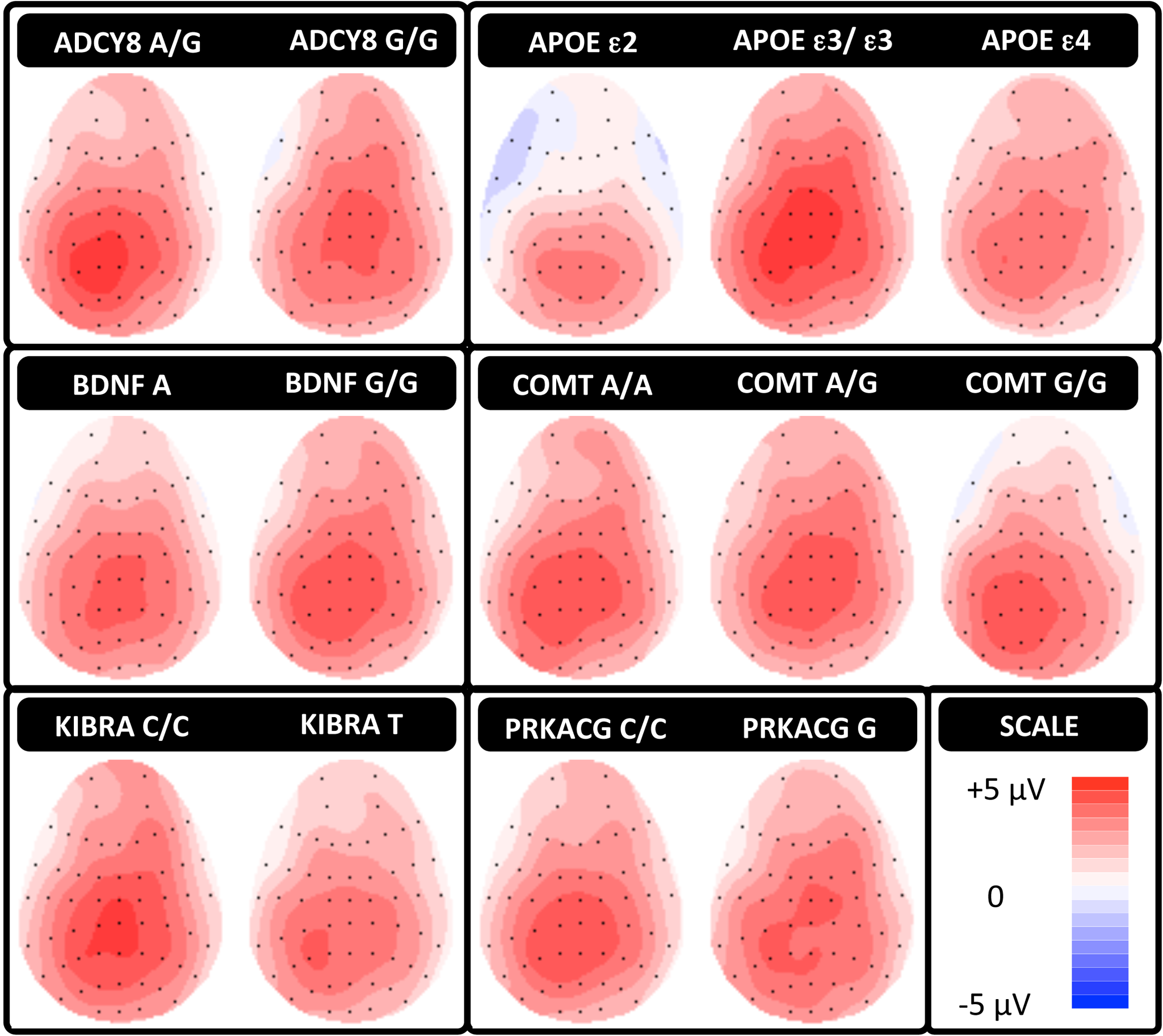

Supplement: Figure S2 — Retrieval related brain activity is unaffected by polymorphisms of ADCY8, APOE, BDNF, COMT, KIBRA, PRKACG. Topographic maps depicting the distributions of the old/new effects (Hits minus CRs) in the 500–700 ms time-window for all genes included in the global omnibus ANOVA that failed to reveal significant genotype differences. As evident from the analysis there is minimal difference between genotypes, with carriers of both common and rare variants of each polymorphism exhibiting the typical left parietal distribution reported in the literature. The scale bar indicates the size of the old/new difference in microvolts. (TIF) [file pone.0098018.s002.tif]
